# Epithelial Cell Adhesion Molecule mRNA Can be a Potential Marker to Predict Metastasis in Hepatocellular Carcinoma Patients

**DOI:** 10.31557/APJCP.2020.21.3.861

**Published:** 2020-03

**Authors:** Iman A Abdelgawad

**Affiliations:** *Department of Clinical Pathology, NCI, Cairo University, Cairo, Egypt. *

**Keywords:** EpCAM, HCC, metastasis, RT-PCR

## Abstract

**Objectives:**

Epithelial cell adhesion molecule [EpCAM] is a surface marker of cancer stem cells that can maintain the capacity for malignant proliferation, invasion, metastasis, and tumor recurrence; hence its detection among hepatocellular carcinoma [HCC] patients may be an important prognostic factor. The aim of this study was to detect EpCAM mRNA expression in the whole blood of HCC patients and normal control subjects to elucidate its clinico-pathological significance among patients with HCC.

**Methods:**

This study was conducted on 74 newly diagnosed HCC patients and forty normal control subjects. Both groups were subjected to the detection of EpCAM mRNA in the whole blood using reverse transcriptase polymerase chain reaction [RT- PCR] technique. EpCAM expression was compared with some of the established prognostic factors of HCC.

**Results:**

EpCAM was detected in 17.5% of the HCC cases and was not expressed in any of the normal control subjects. EpCAM positive cases showed higher serum levels of alpha- feto protein [AFP] and carcinoembryonic antigen [CEA]. Prevalence of EpCAM positivity gave significant results with distant metastasis, lymph node metastasis, and portal vein thrombosis.

**Conclusion:**

EpCAM proved high specificity among HCC patients and its expression was associated with metastasis and portal vein thrombosis. Higher serum levels of CEA among the EpCAM positive patients may attract the attention to a subgroup of HCC patients who are more liable to develop metastasis.

## Introduction

Liver cancer is predicted to be the sixth most commonly diagnosed cancer and the fourth leading cause of cancer death worldwide in 2018. In total, there were 841,080 cases of liver cancer worldwide in 2018 (4.7% of all cancer sites) with 781,631 deaths (8.2% of all cancer deaths) (GLOBOCAN, 2018).

TROP1 and tumor-associated calcium signal transducer 1 are other names for epithelial cell adhesion molecule which is a 30–40 kDa type I membrane protein of 314 amino acids. Mutations in the EPCAM gene have been linked to Hereditary Non-Polyposis Colorectal Cancer, which is a well-known cancer susceptibility syndrome that predisposes to many types of cancer, particularly, colorectal adenocarcinoma and endometrial carcinoma, (Pathak et al., 2019). It is widely expressed on the surface of epithelial cells and epithelial-derived tumor cells, and is important for cell adhesion, proliferation, differentiation, signal transduction, and tissue regeneration (Huang et al., 2018). It is also considered an important marker for hepatic progenitor cells (HPCs), which are thought to have a crucial role in the production new hepatocytes, cell fate decisions , cell migration and differentiation (Atsunori et al., 2019). Only about 35% of HCC cases express EpCAM, although HCC cells are epithelial in origin (Yamashita et al., 2008).

EpCAM has recently been recognized as a surface marker of cancer stem cells (CSCs) (Fouse et al., 2013). EpCAM-positive HCC cells display some of the liver cancer stem cell-like characters which include; self-renewal and differentiation, maintaining the capacity for malignant proliferation, invasion, metastasis, and tumor recurrence (Campbell et al., 2007). These findings indicate that EpCAM may serve in the targeted therapy against cancer which might offer a promising and a novel approach for the treatment of the poor prognosis types of HCC (Ogawa et al., 2014).

Analyzing the circulating tumor cells (CTCs) characteristics in the peripheral blood of cancer patients is both rapid and convenient, which might help in the diagnosis, prognosis prediction, and evaluation of the response to treatment (Slade et al., 2009). 

Occult tumor cells may remain dormant for up to 22 years in the circulation. Later, these cells might cause tumor relapse and metastasis (Meng et al., 2004). Such cells may express different biological characteristics from the primary tumor and may play an important role in tumor recurrence (Stakenborg et al., 2010). CTCs frequently shed into the blood of cancer patients, but in the circulation, most of them usually undergo apoptosis being affected by the immune cells and changes in the microenvironment, or destructed by the shearing forces in the blood vessels. So, only a very small proportion of CTCs can survive, and when given optimum conditions, they may spread and form micro metastases (Mocellin et al., 2006).

HCC recurrence commonly occurs via hematogenous spread (Imamura et al., 2003); which highlights the significance of CTCs detection in predicting HCC recurrence and monitoring of therapy. The greatest challenge of this field is to discriminate the tumor-derived cells from the healthy cells and their content. As most cancers are epithelial in origin, targeting EpCAM therefore became the most commonly used epithelial marker for the capture of CTCs in the blood circulation of carcinoma patients (Antonarakis et al., 2017; Carter et al., 2017).

The aim of this study was to detect the EpCAM mRNA expression in the whole blood of HCC patients and normal control subjects and to elucidate its clinico-pathological significance among patients with HCC. 

## Materials and Methods

This study was conducted on 74 newly diagnosed HCC patients who presented to the outpatient clinic at the National Cancer Institute (NCI), Cairo University over a period of 6 consecutive months from January to June 2014. The sample size was determined by the statistician. All patients who were eligible to the study and were proven to have HCC by their clinical presentation, computed tomography (CT), magnetic resonance imaging (MRI) or by liver biopsy were included. Cirrhosis and PVT are diagnosed by ultrasound or MRI. There were 52 males and 22 females, and their age ranged from 40 to 81 years. Forty apparently healthy volunteers were included as a normal control group. There were 26 males and 14 females, and their age ranged from 37 to 76 years. 

All procedures followed were in accordance with the ethical standards of the responsible committee on human experimentation (institutional and national) and with the Helsinki Declaration of 1975, as revised in 2008 (Yamashita et al., 2008). 

*Inclusion criteria*

All patients with either sex who were confirmed to have HCC were included in the study prior to any therapeutic intervention.

*Exclusion criteria*

Patients with other concomitant malignancies or received any line of cancer therapy were excluded.

*Blood samples from all patients and control subjects were subjected to the following*

1- Liver function tests using Beckman CX9 auto-analyzer.

2- Tumor Markers: AFP and CEA were done using Axsym based on the micro-particle enzyme immunoassay (MEIA) technology.

3- Detection of EpCAM mRNA expression in the whole blood by RT-PCR 

Seven milliliters of whole blood were collected on EDTA containing vacutainers for all patients and control subjects. Isolation of mononuclear cells was performed using Ficoll-Hypaque (Bøyum, 1968). RNA extraction was done from mononuclear cells using InviTrap Spin Blood RNA Mini Kit according to manufacturer’s instructions. The concentration and purity of RNA was determined by measuring its absorbance at 260 nm and 280 nm using nanodrop. Concentrations ranged from (40 – 80 ng/µl) and purity (1.9 – 2.1). One μg RNA was reversely transcribed using high capacity cDNA reverse transcription kit (Applied Biosystems). Reverse transcription was performed in 20 μl reaction containing 1x RT buffer, 0.2mM DNTP, 1x RT random primer, 50U multiscribe TM reverse transcriptase and nuclease free water. The reaction was performed at 25˚C for 10 min, followed by 37˚C for 120 min and 85˚C for 5 min then kept at 4˚C. The reaction for β actin was performed in 25 μl reaction containing 1 μl cDNA, 1x master mix, 25 pmole of each primer F: 5’-GTGGGGCGCCCCAGGCACCA-3’and R: 5’ -CTCCTTAATGTCACGCACGATTTC-3’ (Inoue et al., 1994). The cyclic condition consisted of initial denaturation at 94˚C for 5 min, followed by 30 cycles of denaturation at 94˚C for 1 min, annealing at 63˚C for 2 min and extension at 72˚C for 3 min. Four μl of cDNA was used as template for EpCAM amplification performed in 25 μl reaction containing 1x buffer, 25 pmole of each primer F: 5’- GGACCTGACAGTAAATGGGGAAC -3’and R: 5’- CTCTTCTTTCTGGAAATAACCAGCAC -3’ (Yamashita et al., 2008)., 2U Hot Firepol DNA polymerase [Solis Biodyne], 1X buffer, 1.5mM MgCl_2_ and 200 μM of each deoxynucleotide triphosphate. The cyclic condition consisted of initial denaturation at 95˚C for 15 min, followed by 45 cycles of denaturation at 94˚C for 1min, annealing at 66˚C for 1min, extension at 72˚C for 1 min and final elongation at 72˚C for 7 min. The PCR products were visualized on 2% agarose and were 540 bp for β actin and 186 bp for EpCAM.

*Statistical analysis*

Data analysis was done by using IBM SPSS advanced statistics version 20 (SPSS Inc., Chicago, IL). The descriptive measures were presented in frequency and percentages. For quantitative data, comparison between two groups was done using Mann-Whitney (non-parametric t-test). Chi square test was used to test prevalence of proportions. P-value of ≤ 0.05 was considered significant. 

## Results

Patients’ characteristics are mentioned in Table 1. EpCAM was detected in 13/74 (17.5%) of the HCC cases, while all normal control subjects were negative for EpCAM expression. Distant and lymph node metastases were detected in (19% and 27%) of patients respectively. Thirty % of patients were cirrhotic, 13.5% had portal vein thrombosis, 35% had advanced disease stage (III and IV), while 26% suffered from hepatitis viral infection.

In Table 2, both AFP and CEA showed significantly higher levels among the EpCAM positive cases compared to the EpCAM negative cases (P= 0.022 and 0.024) respectively, while Table 3 shows the comparison of some prognostic factors of HCC according to the positivity of EpCAM expression, which showed significant results with distant metastasis, lymph node metastasis and portal vein thrombosis (PVT).

On comparing cases with CEA levels > 5 ng/ml and cases with AFP levels > 400 ng/ml according to the positivity of EpCAM expression, patients with positive EpCAM expression showed significantly higher levels of CEA (> 5 ng/ml) (P=0.048), while no significant results were detected regarding AFP.

**Table 1:** Patients’ Characteristics

Characteristic		N (74)	Percentage
Age (median and interquartile range)	61 (55 – 67)		
Sex	Males	51	69
	Females	23	31
EpCAM	Positive	13	17.5
	Negative	61	82.5
Stage	I	13	18
	II	35	47
	III	18	24
	IV	8	11
Distant metastasis	Present	14	19
	Absent	60	81
Lymph nodes involvement	Present	20	27
	Absent	54	73
Cirrhosis	Present	22	30
	Absent	52	70
Portal vein thrombosis	Present	10	13.5
	Absent	64	86.5
Hepatitis virus infection	Positive	19	26
	Negative	55	74

EpCAM, Epithelial cell adhesion molecule

**Table 2:** Comparison of AFP, CEA and Liver Enzymes According to the Positivity of EpCAM

		Age (years)	AFP	CEA	AST	ALT	ALP
			( < 10 ng/ml)	(0-3 ng/mL)	(10 – 40units /L )	(7 - 56 units /L)	44 to 147 IU/L)
EpCAM	Neg (61)	60 (54 - 67)	56 (17-189)	5 (3 - 6.4)	56 (33-74)	38 (26-56)	132 (109 - 163)
	Pos (13)	65 (60 – 68)	358 (40 - 673)	6.6 (5 – 30.5)	59 (36-67)	45 (39-54)	152 (146 -166)
	p-value	0.243	0.022*	0.024*	0.949	0.486	0.102

*, Significant; Median and interquartile range in parenthesis; EpCAM, Epithelial cell adhesion molecule; AFP, Alpha-feto protein; CEA, Carcinoembryonic antigen; AST, Aspartate aminotransferase; ALT, Alanine aminotransferase; ALP, Alkaline phosphatase.

**Table 3:** Comparison of Some Prognostic Factors of HCC According to the Positivity of EPCAM

		EpCAM neg (n 61)	EpCAM pos (n 13)	Chi square	P value
Sex	Males	40	11		
	Females	21	2	1.1	0.31
Distant	Present	7	7		
metastasis	Absent	54	6	9.9	0.002*
Lymph node	Present	9	11		
metastasis	Absent	52	2	23.1	<0.001*
PVT	Present	2	8		
	Absent	59	5	26.3	<0.001*
Cirrhosis	Present	15	7		
	Absent	46	6	3.1	0.078
Hepatitis	Present	16	3		
	Absent	45	10	0.01	0.92
Stage	I	11	2		
	II	27	8		
	III	15	3		
	IV	8	0	0	1
	IV	8	0	0	1

*, Significant; EpCAM, Epithelial cell adhesion molecule; PVT, Portal vein thrombosis.

## Discussion

Recent studies have revealed that EpCAM is over-expressed in a variety of human cancers, including HCC (Yamashita et al., 2008). 

Although HCC cells are epithelial in origin, only about 35% of HCC cases express EpCAM (Yamashita et al., 2008). This comes in line with our findings as only 17.5% of patients with HCC were positive for EpCAM expression. On the other hand, all normal control subjects were negative for EpCAM expression, which reflects its high specificity among the cancer patients. Similarly, no healthy or benign liver disease subjects had CTCs detected in a study by Wen Xu et al., (2011). Although CTCs are either not detected or are extremely rare in healthy individuals or patients with benign conditions, there is no sufficient data to describe the incidence of circulating EpCAM-positive epithelial cells commonly associating HCC,as liver injury, cirrhosis, or viral hepatitis (Schulze et al., 2013).

Being easily accessible, CTCs might be considered as promising candidates to predict tumor aggressiveness, recurrence and metastasis (Sun et al., 2013). However, their rarity in the peripheral blood of the patients might limit their clinical utility. It has been proven that EpCAM-positive CTCs may serve as a prognostic marker in HCC after curative resection, as overexpression of EpCAM is significantly associated with the poor clinical outcome of HCC (Sun et al., 2013). 

In this study, among the EpCAM positive subjects, 7/13 (54%) had distant metastasis, 11/13 (85%) had LN metastasis, and 8/13 (61.5%) had PVT. The prevalence of positivity of EpCAM expression gave significant results when compared to these 3 prognostic factors, but not with the disease stage or other tested prognostic factors which is contradictory to the results detected by other investigators who found that EpCAM-positive cancer patients commonly exhibit an advanced tumor stage (Sun et al., 2013; Anthony et al., 2014). 

Serum alpha-feto protein (AFP) is commonly used for diagnosis and surveillance of HCC and has been suggested as an independent indicator for prognosis as HCC patients with a high serum AFP levels tend to have shorter survival (Llovet et al., 2012). However, it lacks sufficient sensitivity and specificity, as about 30% to 40% of HCC patients are AFP negative, and AFP mRNA was reported to be detected in patients with cirrhosis or chronic hepatitis B infection (Aselmann et al., 2001). RT-PCR for AFP or albumin has been used to detect CTCs among HCC patients (Ijichi et al., 2002); however, results are inconsistent due to their low specificity.

It has been detected that EpCAM-positive CTCs are associated with bad prognostic criteria; as high AFP, presence of vascular invasion, and poor differentiation (Yamashita et al., 2013). These findings support the potential role of CTCs in prognostic stratification of patients and treatment decision-making among patients with HCC, which has been challenged before by the great prognostic heterogeneity of this disease (Kelley et al., 2013).

Supporting these findings, we detected higher serum levels of AFP among the EpCAM positive cases compared to the EpCAM negative cases (P = 0.022). Five out of 13 (38%) of the EpCAM positive cases, had AFP levels > 400 ng/dl.

Carcinoembryonic antigen (CEA) is an immunoglobulin superfamily cell surface glycoprotein that might play a role as an adhesion molecule (Jessup et al., 1989). Since alterations in cell adhesion are believed to be involved in cancer invasion and metastasis, it was further suggested that CEA may play a role in these processes (Jessup et al., 1989).

EpCAM has been reported to promote tumor formation and metastasis either by disrupting the link between a-catenin and F-actin (Winter et al., 2003) or by facilitating the immune escape of tumor cells (Winter et al., 2003). This supports the significant results between prevalence of EpCAM expression and the presence of both distant metastasis and lymph node metastasis in our study. Similarly, CEA is thought to play a role in the metastatic processes (Jessup et al., 1989). This can explain our findings that EpCAM positive cases showed higher serum levels of CEA compared to EpCAM negative cases (P = 0.024). It has also been detected that 10/13 (77%) of the EpCAM positive cases had CEA serum levels higher than 5 ng/ml. Six out of these 10 patients (60 %) had concomitant distant metastasis. This can take us to the assumption that the elevation of both EpCAM and CEA in HCC patients may give a strong indicator for the presence of metastasis.

The prevalence of EpCAM expression positivity also gave significant results with portal vein tumor thrombosis (PVT), and 80% of the patients having PVT were found to be EpCAM positive, from whom 50% had CEA levels > 5 ng/ml. 

Supporting our results, Xu et al., (2011), detected higher positivity rate and number of detected CTCs in HCC patients with PVT compared to those patients without PVT, which suggests that PVT may also be a source of systemic spread of CTCs contradicting the previous assumption that PVT would mainly cause intrahepatic rather than distant metastases (Mitsunobu et al., 1969). 

EpCAM is absent in blood cells , not expressed on mature hepatocytes and is expressed in only approximately 35% to 60% of HCC tumors by immunohistochemistry or PCR-based techniques (Kelley et al., 2013).

It is possible that non-EpCAM-expressing HCC cells exist in circulation and are undetectable by the current technologies, which may account for our inability to detect CTCs in some of our HCC patients. Adding to this, during epithelial mesenchymal transition EMT, down regulation of EpCAM is required for the invasion by cancer cells. EMT is a process via which epithelial cells gain mesenchymal characteristics that enhance their migratory capacity and invasiveness, and increase their resistance to apoptosis (Kalluri et al., 2003). 

In addition, the upregulation of EpCAM expression due to regenerative activity during inflammatory reactions (Kalluri et al., 2003) which raises the possibility that upregulation of EpCAM expression in HCC may be partially due to the inflammatory response accompanying the underlying cause of HCC as liver cirrhosis. Collectively, all these data suggest that CTC isolation and detection in HCC may require combining EpCAM with other markers like CD90, and CD133 which have been used to identify potential hepatic CSCs (Ma et al., 2007). In this study we didn’t examine for the presence of theses 2 markers, which we recommend for further studies.

This study has some limitations; confirmation of EpCAM positivity by immunocytochemistry was needed to give more precise information, also follow up of EpCAM positive cases for long durations was needed to detect the impact of EpCAM on the patients’ survival and tumor metastasis. Further studies will be done to fulfill all the limitations encountered in this study.

In conclusion, EpCAM mRNA was detected in the whole blood of 17.5% of the HCC patients, while none of the normal control subjects were positive for EpCAM expression. This reflects the high specificity of EpCAM among cancer subjects. Detection of CTCs in the whole blood of HCC patients is a non-invasive procedure that can give a clue about the prognosis and treatment strategies for the HCC patients.

Higher serum levels of CEA among the EpCAM positive patients may attract the attention to a subgroup of HCC patients who carry poor prognosis and are more liable to develop metastasis and recurrence. Such group of patients may be the target for further studies to benefit from certain targeted therapies.

Further studies with larger sample sizes are recommended to evaluate the efficacy of EpCAM as a diagnostic and prognostic marker for HCC. 

Quantitation of the CTCs may give more accurate results especially if meant to be used to evaluate the efficacy of treatment. Since tumor malignancy is associated with complex signaling pathways; it is desirable that CTCs be used in combination with another index, to monitor their clinical relevance. 

## Abbreviations

HCC: Hepatocellular carcinoma

EpCAM: Epithelial cell adhesion molecule

CSCs: Cancer stem cells

CTCs: Circulating tumor cells

CT: Computed tomography

MRI: Magnetic resonance imaging

MEIA: Microparticle enzyme immunoassay

AFP: Alpha-feto protein

CEA: Carcinoembryonic antigen

PVT: Portal vein thrombosis
